# Supplementary material for: Catalytic Methanation over Natural Clay-Supported Nickel Catalysts
Source: Molecules. 2025 May 9;30(10):2110. doi: 10.3390/molecules30102110 (PMC12113879; doi:10.3390/molecules30102110)
Supplement: Supplementary file 1 [file molecules-30-02110-s001.zip › molecules-3544526-supplementary.pdf]

Supplementary Materials

Catalytic methanation over natural clay supported nickel catalyst

Alejandra Cue Gonzalez[1], Elsa Weiss-Hortala[1], Quoc-Nghi Pham[2], Doan Pham Minh[1,3,*]

[1]*Université de Toulouse, IMT Mines Albi, UMR CNRS 5302, Centre RAPSODEE, Campus Jarlard, F-81013 Albi, cedex 09, France.*

[2]*Université Paris-Saclay, CNRS, ICMMO, Orsay 91405, France*

[3]*Sustainable Environment Research Institute, Chulalongkorn University, Bangkok, 10330, Thailand*

[*]Corresponding author: doan.phamminh@mines-albi.fr. Tel. +33 563493258

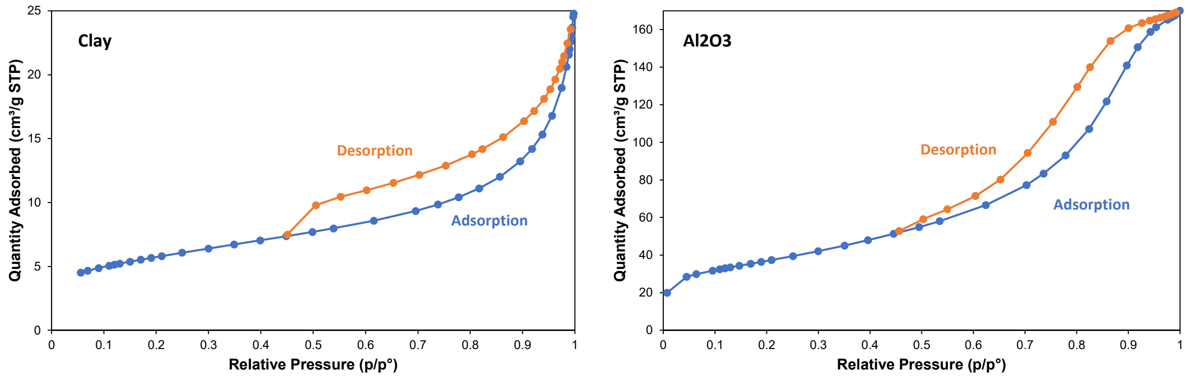

Figure S1. Nitrogen adsorption-desorption isotherms of the initial clay and Al_2O_3 supports.

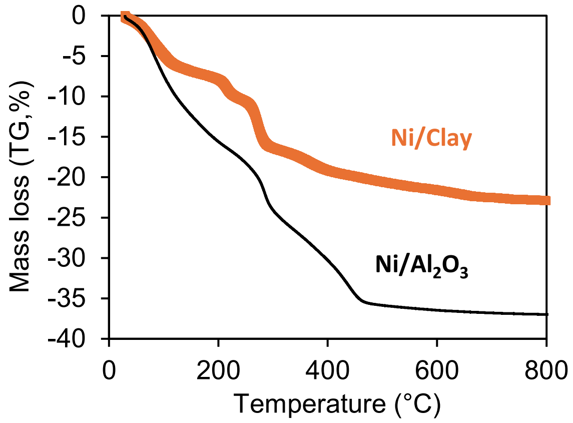

Figure S2. TG curves under the air atmosphere of the dried catalysts, showing the different dehydration phenomena of the natural clay and the alumina hydrate, and the thermal decomposition of the nickel precursor (Guggenheim et al., 1987), (Keely & Maynor, 1963).

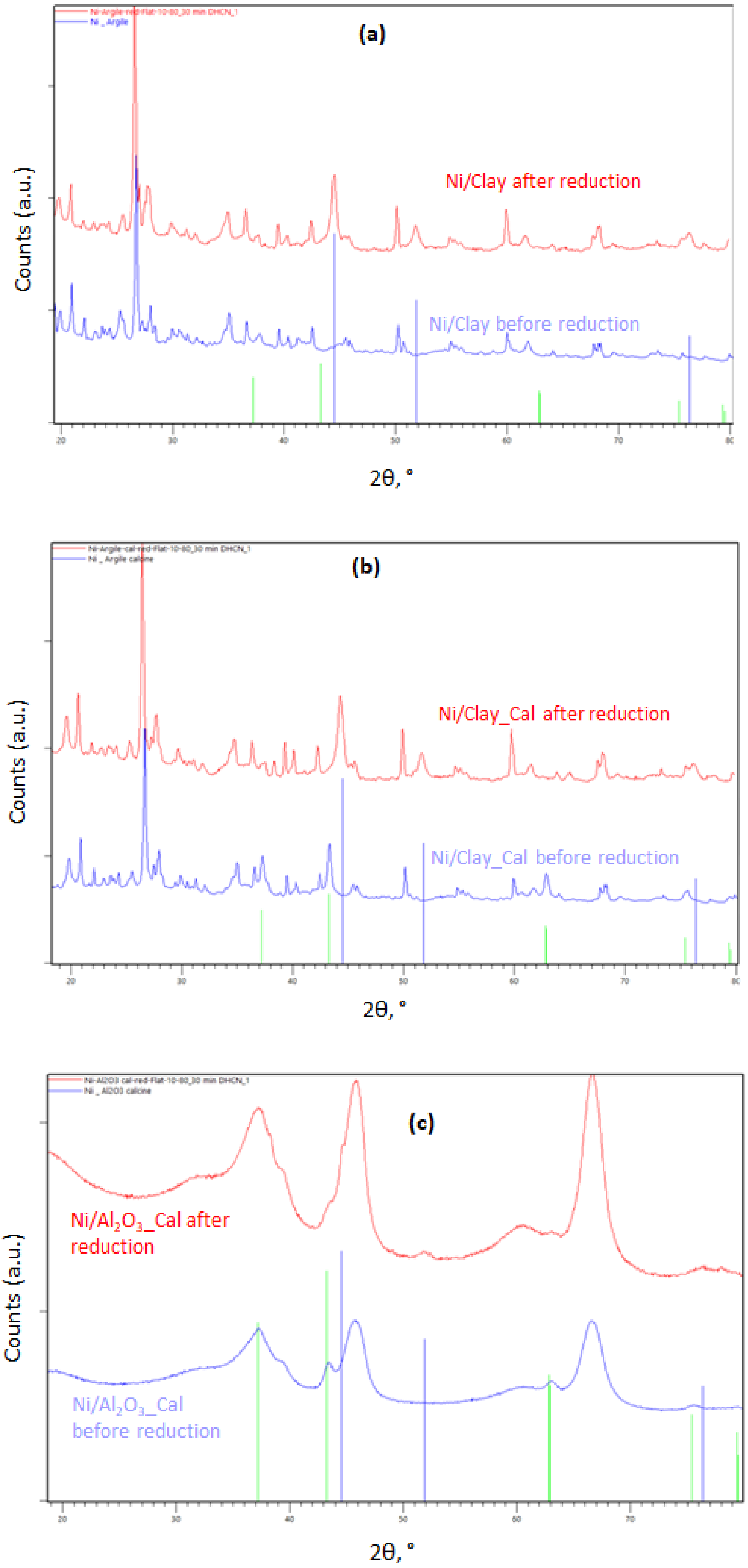
(a)
Ni/Clay after reduction
Ni/Clay before reduction
Counts (a.u.)
2θ, °
(b)
Ni/Clay_Cal after reduction
Ni/Clay_Cal before reduction
Counts (a.u.)
2θ, °
(c)
Ni/Al2O3_Cal after reduction
Ni/Al2O3_Cal before reduction
Counts (a.u.)
2θ, °

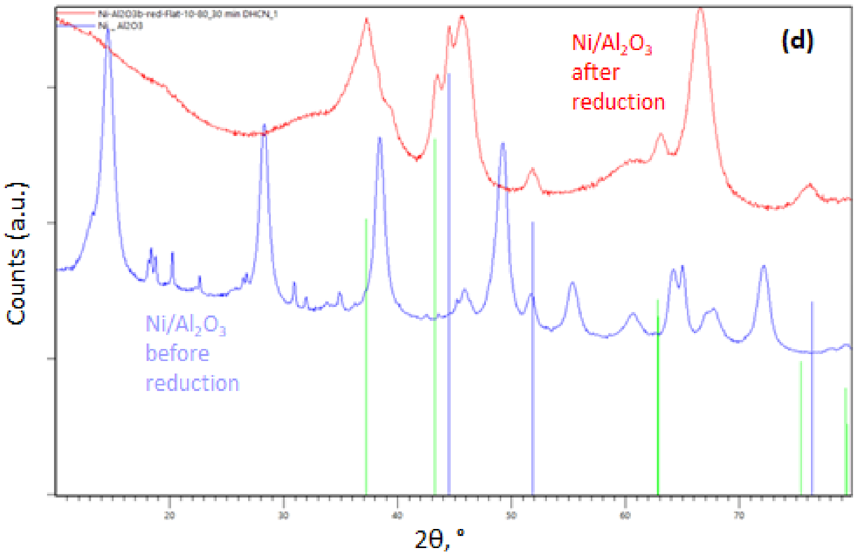

Figure S3. Comparison of XRD patterns of the catalysts before and after reduction under hydrogen at 500 °C: (a): Ni/Clay; (b): Ni/Clay_Cal; (c): Ni/Al_2O_3; (d): Ni/Al_2O_3_Cal. Vertical purple sticks: standard pattern of metallic nickel (peaks at 44.5, 51.8 and 76.3 degrees); vertical green sticks: standard pattern of NiO (peaks at 37.2, 43.3, and 62.8 degrees).

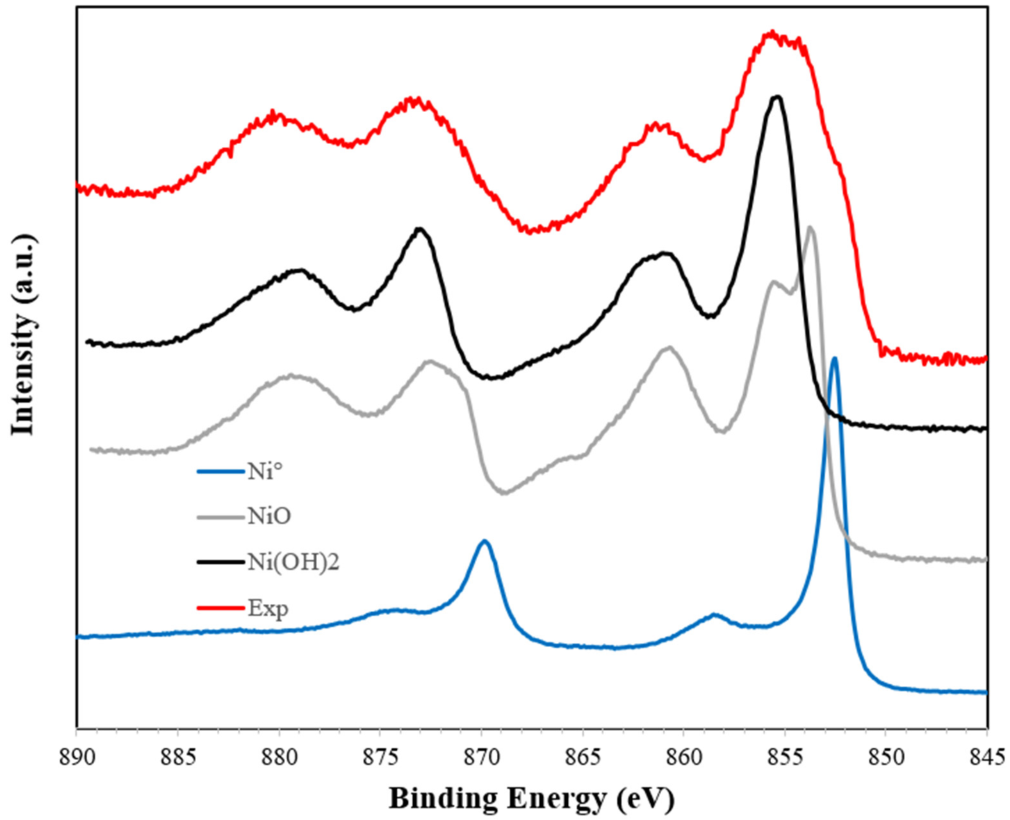

Figure S4. Comparison of Ni 2p XPS spectra recorded under identical experimental conditions for standard compounds (metallic Ni, NiO, and $Ni(OH)_2$) and the reduced Ni/Clay catalyst.

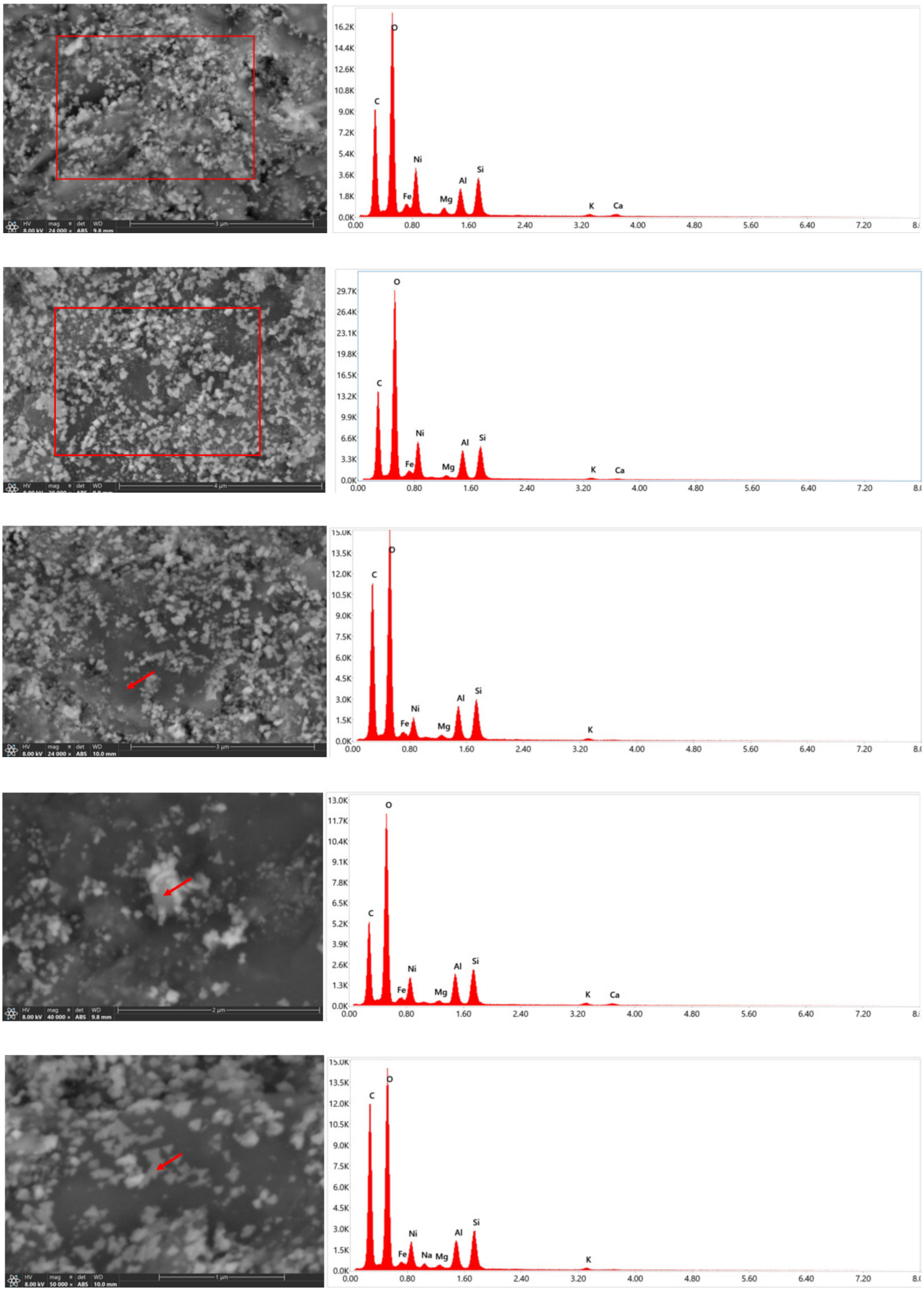

Figure S5.SEM-EDX analyses of the Ni/Clay catalyst, which highlighted the presence of minerals on the surface of the Ni/Clay catalyst.

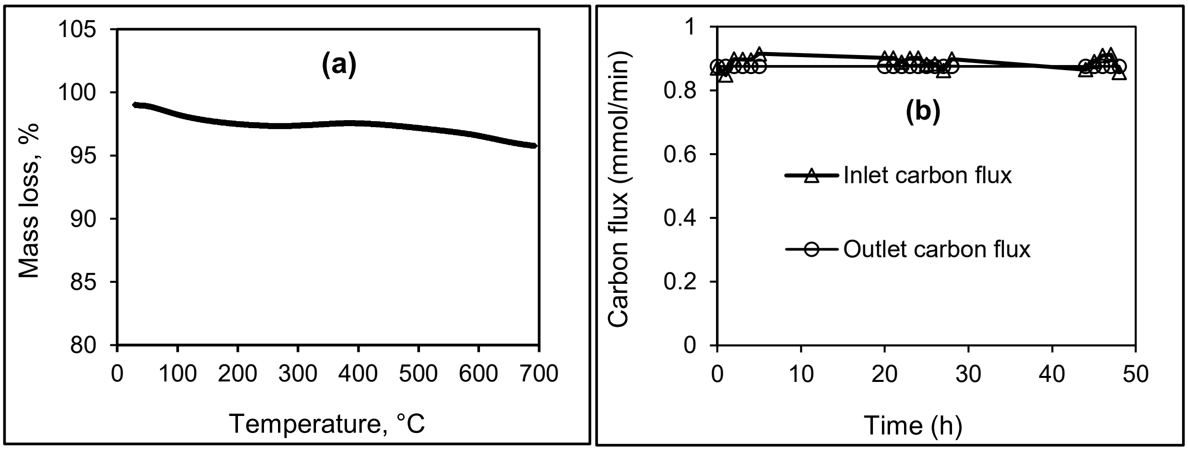

Figure S6. TG analysis (a) and carbon balance (b) in the methanation of the synthetic biogas over Ni/Clay for a long reaction time; reaction conditions: in-situ reduction at 500 °C, reaction temperature at 500 °C, 500 mg catalyst, inlet gas flowrate: 22.0 mL/min CO_2, 90 mL/min H_2, 3.75 mL/min N_2, and 33.75 mL/min CH_4; WHSV = 17940 $mL{\cdot}g_{cat}^{-1}{\cdot}h^{-1}$.

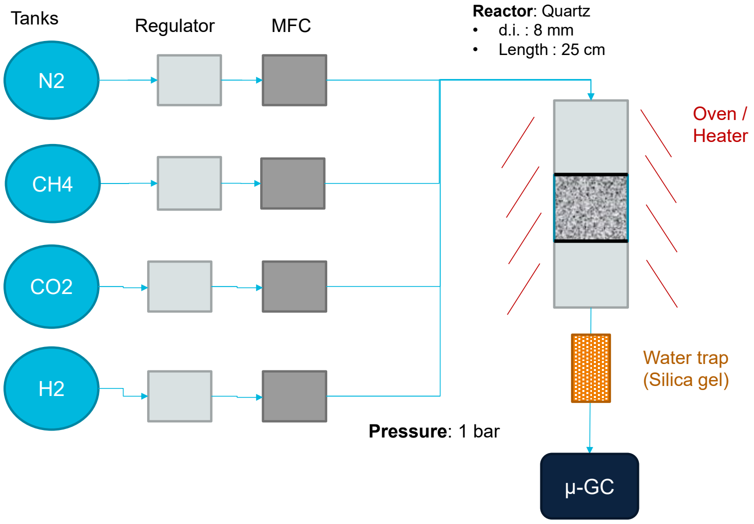

Figure S7. Schematic of the reactor system.

References

Guggenheim, S., Chang, Y.-H., & Koster van Groos, A. F. (1987). Muscovite dehydroxylation; high-temperature studies. *American Mineralogist*, *72*(5–6), 537–550.

Keely, W. M., & Maynor, H. W. (1963). Thermal Studies of Nickel, Cobalt, Iron and Copper Oxides and Nitrates. *Journal of Chemical & Engineering Data*, *8*(3), 297–300. https://doi.org/10.1021/je60018a008
